# New York College of Dentistry

**Published:** 1882-05

**Authors:** 


### NEW YORK COLLEGE OF DENTISTRY.

The sixteenth annual commencement.

The number of matriculates for the session was one hundred and twenty-four.

The degree of D.D.S. was conferred on the following persons by Dr. Wm. H. Allen, President of the Board of Trustees:

| NAME. | RESIDENCE. | NAME. | RESIDENCE. |
|---|---|---|---|
| Charles P. Adams | Massachusetts. | Harry C. Medcraft | Connecticut. |
| Martin L. Ballard | Ohio. | Karl J. Milke | Germany. |
| Ruby E. Clifford | England. | William H. Mitchell | New Jersey. |
| Alfred Dennis | New York. | Oscar L. Moser | Germany. |
| Frank S. Derby | New York. | Archibald McFadgen | California. |
| Charles A. DuBois | Denmark. | Benjamin C. Nash | England. |
| John H. Feindel | New York. | James Neil, Jr. | New York. |
| Anthony A. Formel | Cuba. | Charles E. H. Phillips | Connecticut. |
| Edward L. Fuller | Massachusetts. | Willis A. Reeve | New York. |
| Fred. W. Gillen | New York. | Richard Shuebruk | England. |
| Martin C. Gottschaldt | Germany. | Roswell O. Stebbins | California. |
| Addison H. Griffing | New York. | Albert J. Stillman | New York. |
| F. L. Hesse, M.D. | Germany. | Augustus A. Syme | Connecticut. |
| Alex. Kronmeyer | Cent. America. | Frederick J. Wells | New Jersey. |
| Eduardo Lopez | Ecuador, S. A. | John C. Westervelt | New Jersey. |

